# Decopy: detect and correct with pinyin for Chinese spelling correction

**DOI:** 10.1098/rsos.250426

**Published:** 2025-09-24

**Authors:** Zijian Zhang, Jinfeng Yuan

**Affiliations:** ^1^ College of Computer Science, Chongqing University, Chongqing, People’s Republic of China

**Keywords:** Chinese spell correction, natural language processing, large language models, detection-correction framework

## Abstract

Chinese spelling correction (CSC) is a critical and complex task focused on detecting and correcting spelling errors in Chinese text. Previous research has been hampered by issues such as misleading error information, over-reliance on high-frequency characters and scarcity of training data. This article proposes *Decopy*, a novel CSC model that employs an advanced detection-correction framework and an innovative error masking strategy incorporating pinyin features. Decopy not only captures semantic information (word embeddings) and positional information (position embeddings) but also recognizes phonetic features (pinyin embeddings). By leveraging phonetic information directly from the pinyin level, Decopy minimizes the reliance on confusing elements and reduces misleading information. To address the scarcity of training data, we constructed a new CSC dataset based on THUCNews for pre-training Decopy. This enhances Decopy’s comprehensive understanding of the input information, particularly additional pinyin information. The experimental results on SIGHAN15 and three domain-specific datasets—LAW, medical and official document writing—demonstrate that Decopy achieves significant improvements and outperforms previous state-of-the-art methods. Furthermore, we evaluate the performance of several high-performance large language models in the CSC task to assess their capabilities in this field.

## 1. Introduction

**Figure 1.**
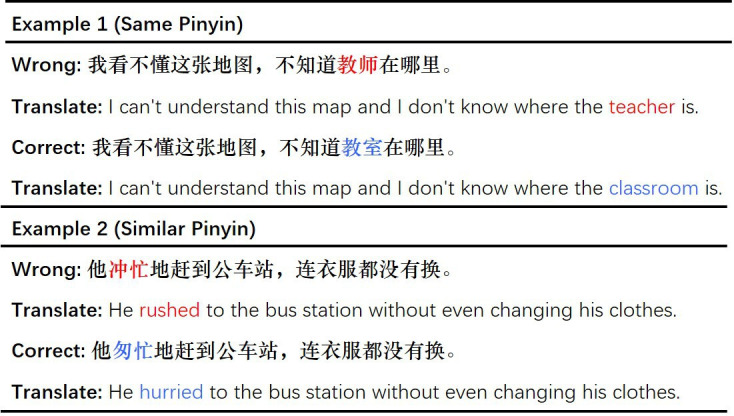
The examples of Chinese spelling errors.

Chinese spelling correction (CSC) is a crucial task that plays a pivotal role in various natural language processing (NLP) applications, including search query correction [1,2], optical character recognition (OCR) [3] and automatic essay scoring [4]. Spelling errors in Chinese are common in daily life and primarily stem from human writing, input method editor (IME) typing, automatic speech recognition (ASR) and OCR. Figure 1 shows several examples of spelling errors. However, the CSC task is highly challenging, requiring human-level natural language understanding ability [5–7]. Unlike in English, Chinese words lack delimiters and can consist of a single character or multiple characters. Furthermore, Chinese comprises over 85 000 characters, in contrast to the 26 letters in the English alphabet, and the semantics of each character can vary significantly depending on the context.

In recent years, significant progress [8,9] has been made in this task with the assistance of pre-trained language models. The majority of these studies employ BERT [10] as the backbone model and further fine-tune it to adapt to the CSC task. Leveraging the powerful linguistic capabilities of BERT, the state-of-the-art performance has been further advanced. To further mitigate the overcorrection problem, recent works have explored novel training strategies and task formulations. For example, Liu *et al.* [11] challenged the traditional sequence tagging paradigm by reframing CSC as a sentence rephrasing task, compelling the model to correct errors based on sentence-level semantics instead of local character mappings. Wei *et al.* [12] proposed a prior-knowledge-guided teacher-student framework, which guides the model to focus more on language modelling rather than merely memorizing error patterns. However, these studies encountered several common issues. *First*, misdirection from erroneous information. BERT uses a bi-directional transformer structure, which provides the model with all contextual information. This feature may produce side effects when the input text contains misspelled characters. *Second*, over-reliance on high-frequency characters. When there are multiple candidate characters for a masked position, BERT prefers to choose the most frequent one in the training datasets, without considering the connection with the context. This preference will increase overcorrections since the model tends to overcorrect those correct but infrequent characters. *Third*, a scarcity of training data. The current CSC training sets are very sparse and limited in size. Most previous methods directly fine-tune BERT using a relatively small 271 k dataset, the majority of which contains errors generated by automated tools rather than human-generated errors. BERT is unable to fully learn the features of the CSC task with this dataset alone.

In this article, we propose *Decopy* to address these gaps by dividing the CSC task into two sub-tasks: error detection and correction. The detection network is responsible for predicting the probability of word errors at different positions in the text. In the correction network, in order to enhance the model’s ability to understand sentences with spelling errors, the structure of BERT is modified by adding pinyin information to the embedding layer, and a masked language model with pinyin features is trained to predict the masked positions. To address the scarcity of training data, we construct a large-scale CSC corpus by extending the THUCNews dataset [13], which facilitates robust pre-training. Based on previous research, we conduct experiments using the widely used benchmark dataset SIGHAN15 [14]. Meanwhile, we use the domain-specific dataset ECSpell [15] to evaluate the performance of Decopy on domain-specific CSC. The experiments demonstrate that Decopy can effectively alleviate the above problems and achieve state-of-the-art performance. In addition, benefiting from the outstanding ability and performance of large language models in NLP tasks [16–18], we test and analyse the effect of several relatively new LLMs with strong performance on the CSC task.

In summary, our contributions are as follows:

— We propose a novel CSC method called Decopy, which employs an innovative detection-correction framework that incorporates a pinyin feature masking strategy. This approach effectively mitigates misdirection caused by erroneous information and reduces over-reliance on high-frequency characters.
— We construct a large-scale, high-quality training corpus specifically designed for the CSC task to address the scarcity of training data.
— Comprehensive experiments conducted on the SIGHAN15 benchmark and three domain-specific datasets validate the superior performance of our proposed method.
— We systematically evaluate the capability of several state-of-the-art LLMs in the CSC task to assess their capabilities in this field.

## 2. Related work

### 2.1. Early work

The Chinese spelling check is a fundamental NLP task that has been researched for over 30 years since the early 1990s. Early studies in this area depended on manually designed rules [19]. Subsequently, conventional machine learning algorithms [20] were introduced into this domain. Given a sentence, the language model’s perplexity first detects error positions. Candidates for corrections can then be generated based on character similarity, which is commonly accomplished using a confusion set.

### 2.2. BERT in Chinese spelling correction

In the era of deep learning, some studies have attempted to employ models based on Recurrent Neural Network‌ (RNN) or Long Short-Term Memory‌ (LSTM) structures for CSC tasks. However, these models suffer from information loss when handling long sequences. Many novel CSC approaches have emerged following the proposals of the transformer [21] and the pre-trained language model BERT [10]. These studies fine-tuned BERT-based models using CSC training data and confusion sets. Some studies have modified the BERT structure to integrate the pronunciation and shape information of the input characters.

### 2.3. Detection-correction framework for Chinese spelling correction

Recently, a new detection-correction framework has been proposed. To avoid the noise caused by spelling errors, previous studies have attempted to add an additional layer for error detection. Some of these studies, such as BERT_CRS [22], directly utilized the sequence output of the final BERT layer for binary classification. Other studies, such as soft-masked BERT [8] and MDCSpell [23], have introduced additional structures for detection. Nevertheless, these methods have some side effects and still fail to resolve the issue of misdirection caused by incorrect characters. This inspired us to develop an advanced framework.

## 3. Methodology

### 3.1. Problem formulation

Chinese spelling checking can be formalized as follows: given a text sequence of n Chinese characters $X=\left(x_{1},x_{2},\ldots ,x_{n}\right)$*,* the goal is to output $Y=\left(y_{1},y_{2},\ldots ,y_{n}\right)$*,* where *X* represents the original text containing some error characters and *Y* represents the correct text after correction. *X* and *Y* have the same length.

### 3.2. Model framework

**Figure 2.**
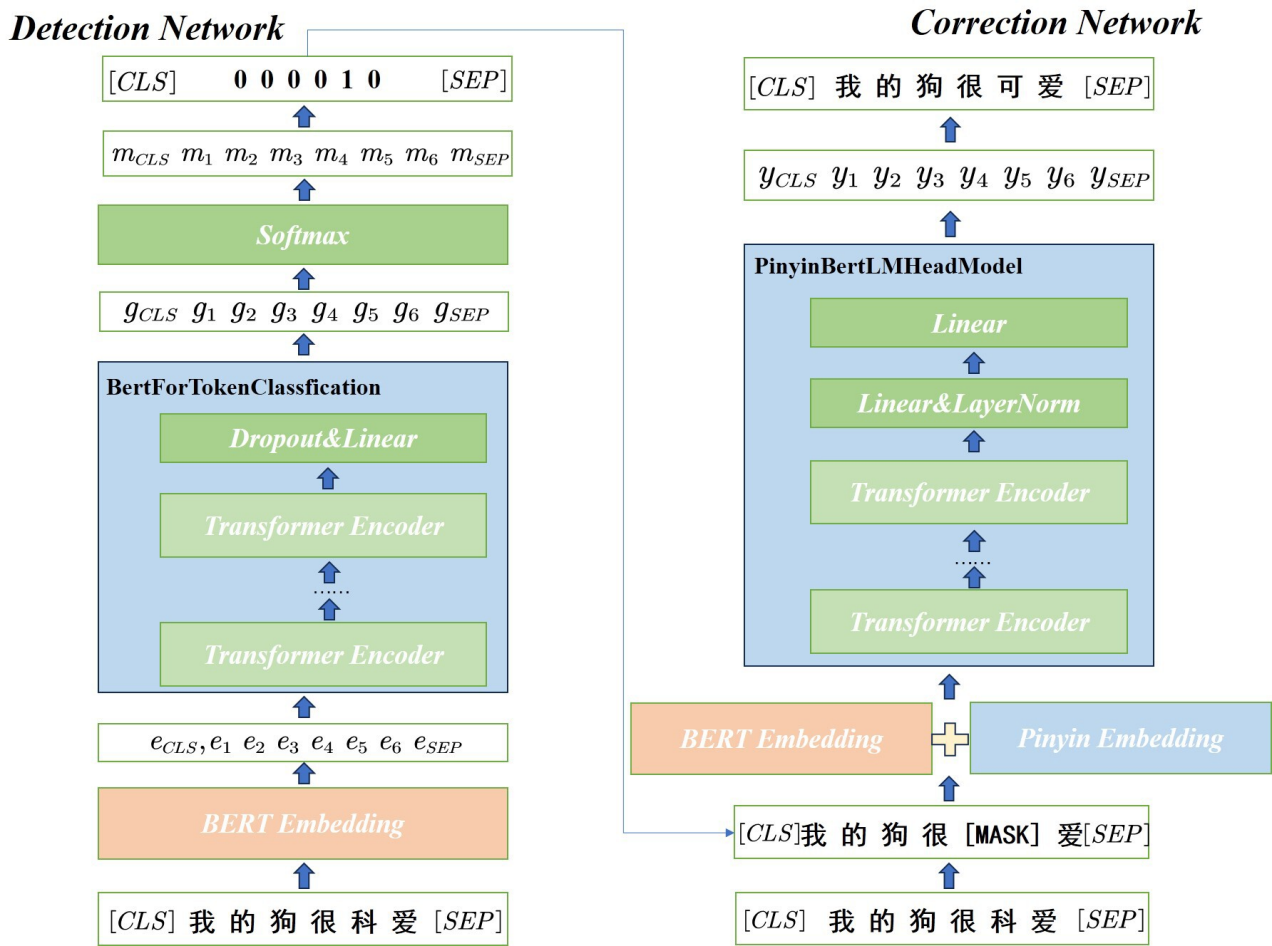
The overall architecture of Decopy.

In this study, we propose a novel CSC method called Decopy, specifically designed to address the key challenges of misdirection, overcorrection and data scarcity. As illustrated in figure 2, the core of Decopy is a two-stage framework consisting of a detection network and a correction network.

The first stage, referred to as the detection network, aims to reduce misdirection caused by erroneous information. Rather than directly feeding a potentially noisy sentence into the correction module, this network first classifies each input token as correct or incorrect. This preliminary filtering step identifies high-confidence error candidates, thereby improving the accuracy of subsequent corrections.

In the second stage, the correction network addresses the model’s tendency to over-rely on high-frequency characters and further minimizes misdirection through two key mechanisms. First, we implement an innovative error masking strategy in which the identified error tokens are masked within the embedding sequence. This compels the correction network to rely on contextual information rather than the misleading character itself. Second, the correction network is augmented with an integrated pinyin embedding mechanism, which introduces phonetic guidance. This enables the model to select corrections that are both contextually and phonetically appropriate, thereby avoiding the selection of statistically frequent but incorrect characters.

### 3.3. Detection model

We consider error detection to be a binary classification problem, where each token has only two possible states: correct or incorrect. The detection model aims to determine the state of each token.

The model is based on the BERT architecture and consists of 12 identical transformer encoders internally. Each encoder contains a multi-head self-attention operation followed by a feedforward network, defined as follows:


(3.1) $$\text{MultiHead}=\text{Concat}\left({\text{head}}_{1},\ldots ,{\text{head}}_{n}\right)W^{O},$$

(3.2) $${\text{ head }}_{i}=\mathrm{Attention}\left(QW_{i}^{Q},KW_{i}^{K},VW_{i}^{V}\right),$$

(3.3) $$\mathrm{FFN}(X)=\max\left(0,XW_{1}+b_{1}\right)W_{2}+b_{2}.$$

Here, Q, K and V, respectively, represent the current input sequence’s representation, either as character embeddings or the output of the preceding Transformer block. The hidden states sequence at BERT’s final encoder is denoted as $H_{detect}=\left(h_{1},h_{2},\cdots \;,h_{n}\right)$.

The model’s output format is obtained from equation (3.4), where $G_{detect}=\left[g_{1},g_{2},...,g_{n}\right]$, for the *i*th token in the sequence, $g_{i}=\left[g_{i,0},g_{i,1}\right]$ represents the scores for whether the token belongs to class 1 (error) or class 0 (correct).


(3.4) $$G_{detect}=\mathrm{FFN}(H).$$

To obtain the error probability of each token, a softmax function is used to process the output of the model. The specific expression is as follows:


(3.5) $$p_{i}=softmax(g_{i}),$$

where $p_{i}$ is a binary tuple that includes the probabilities of the tokens belonging to the error class and the correct class. Notably, $p_{i,1}$ represents the error probability of the *i*th token, with higher values indicating a higher likelihood that the token is incorrect. During the error detection process, a threshold θ (ranging from 0 to 1) is crucial for identifying error tokens, reflecting the tolerance for errors. A lower threshold increases the risk of overcorrection, while a higher threshold increases the risk of undercorrection.

### 3.4. Correction model

The error correction network is constructed based on the BERT model. In this process, the original sentence is re-encoded into embedding vectors and combined with the mask sequence $M=\left(m_{1},m_{2},...,m_{n}\right)$*,* generated using an error masking strategy. The character sequence $Y=\left(y_{1},y_{2},...,y_{n}\right)$ output by the model represents the prediction results of the error correction network.

**Figure 3.**
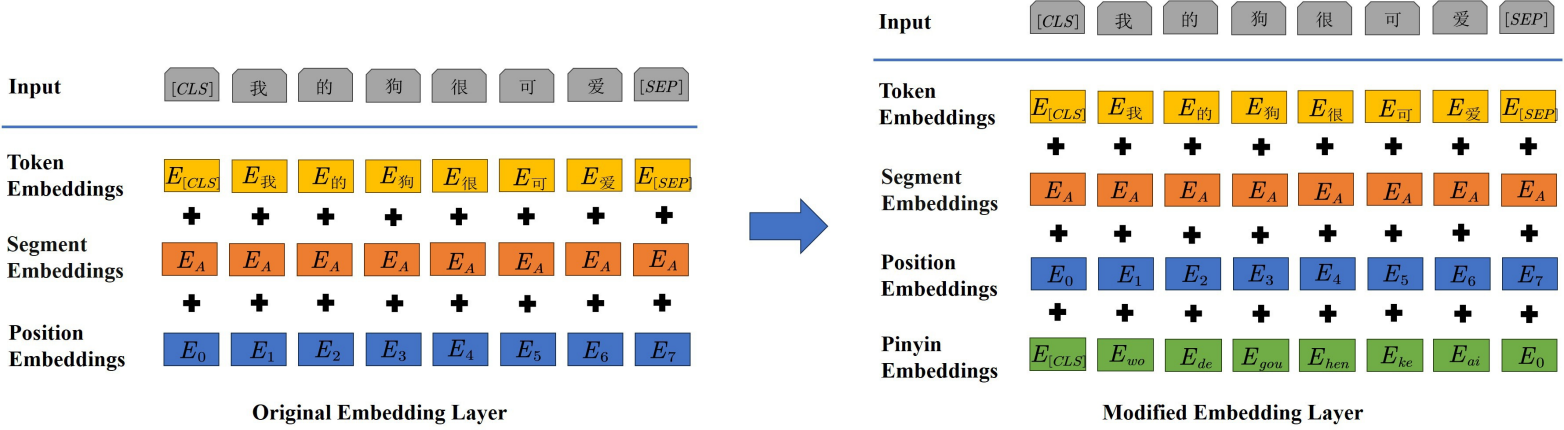
The embedding layer.

*Pinyin embedding*. This study innovatively modifies the embedding layer of the BERT model by introducing pinyin embedding. The original and modified embedding layers are shown in figure 3. After modification, the correction model can obtain the representation $e_{i}$ of each character by


(3.6) $${e_{i}}^{\prime }=e_{i}+py_{i},i=1,2,\ldots ,n,$$

where $e_{i}$ is the original BERT embedding of $x_{i}$, and $py_{i}$ is the pinyin embedding of $x_{i}$. In this representation, by employing a weighted merging of the original embedding and pinyin embedding, the model effectively integrates semantic information and pinyin information. This integration enables the model to achieve a richer and more detailed representation of each character.

*Error masking strategy*. For each token in the sequence, the error probability $p_{i,1}$ output by the detection network will be compared with the set threshold θ. Based on the comparison results, a masking sequence $M=\left(m_{1},m_{2},...,m_{n}\right)$ will be generated. The rule for generating the mask is expressed as follows:


(3.7) $$\begin{array}{r} m_{i}=\left\{ \begin{array}{ll} e_{mask}+py_{i} & \text{if }p_{i,1}>\theta \\ {e_{i}}^{\prime } & \text{otherwise} \end{array}\right. \end{array}.$$

If the error probability is less than or equal to θ, then the token will remain unchanged. If the error probability exceeds the θ, then the token should be masked. The original embedding will be replaced with the embedding of [MASK], whereas the pinyin embedding will be retained. The purpose of this strategy is to minimize misleading information and maximize the useful pinyin information.

*Output*. The error correction network predicts a result for each token, and the probability of token $x_{i}$ being corrected to $y_{i}$ can be expressed as


(3.8) $$P(y_{i}=j|x_{i},C)=softmax(Wh_{i}+b),$$

where $P(y_{i}|x_{i},C)$ represents the probability of correcting $x_{i}$ to j given context C and incorrect vocabulary $x_{i}$, $h_{i}$ represents the output of the last hidden layer, and $softmax(Wh_{i}+b)$ evaluates the probability of correcting the incorrect vocabulary $x_{i}$ to j in context C through a linear layer and an activation function.

### 3.5. Learning

The learning of the detection and correction model is conducted separately, each with a distinct optimization objective as follows:


(3.9) $$L_{detect}=-\sum_{i}(y_{i}\log({\hat{y}}_{i})+(1-y_{i})\log(1-{\hat{y}}_{i})),$$

(3.10) $$L_{correction}=-\sum_{i=1}^{N}\sum_{c=1}^{C}y_{ic}\log({\hat{y}}_{ic}),$$

where $L_{detect}$ is the objective function of the detection network, and $L_{correction}$ is the objective function (and final decision) of the correction network. In equation (3.9), $y_{i}$ is the ground-truth label and ${\hat{y}}_{i}$ is the predicted probability of the token being an error. In equation (3.10), N and C denote sentence length and vocabulary size, respectively. $y_{ic}$ is a binary indicator, which is 1 if the correct character for position i is the *c*th character in the vocabulary, and ${\hat{y}}_{ic}$ is the predicted probability for that character. As our models are trained independently, there is no need to linearly combine the objective functions.

### 3.6. Training corpus

As identified in the introduction, the scarcity of high-quality training data is a key bottleneck in CSC research. Existing datasets are often limited in scale and may not cover a diverse range of errors. To overcome this limitation, we construct a large, high-quality training corpus for CSC based on the THUCNews dataset [13]. Specifically, we segment the original corpus into clauses and perform a series of data cleansing tasks to meet the requirements of CSC, such as restricting text length, converting between simplified and traditional Chinese, correcting punctuation, handling special symbols and annotating errors that are difficult to recognize. Then, we generate errors by replacing some tokens with characters in the confusion set that have a similar pinyin. Following these procedures, we obtained a total of 2.51 million pre-training data.

Since pre-training BERT with similar tasks has been demonstrated to improve the integration of external information into BERT [8,24,25], we pre-trained all our models with this dataset to improve their comprehension of input information, particularly additional pinyin information.

## 4. Experiments

### 4.1. Datasets and metrics

*Datasets.* The fine-tuning dataset includes 10 000 manually annotated samples from SIGHAN15 [14] and 271 000 automatically generated samples provided by Wang *et al.* [26]. Decopy is fine-tuned using this dataset with a batch size of 32 and a learning rate of 2e−5. Furthermore, to evaluate Decopy’s performance on domain-specific CSC, we fine-tune Decopy using LAW, medical (Med) and official document writing (ODW) datasets from ECSpell [15], resulting in models for three specialized domains: Decopy-Law, Decopy-Med and Decopy-Odw.

*Evaluation metrics.* We evaluate the sentence-level detection and correction performance using Precision, Recall and F1 score. A sentence is considered to be processed correctly only if all the errors are successfully detected or corrected. The F1 score is a crucial indicator because it comprehensively reflects the performance of a method. We conduct experiments on both the widely used benchmark dataset SIGHAN15 [14] and the domain-specific dataset ECSpell [15].

### 4.2. Baseline methods

We compare Decopy with the following typical baseline methods. We report the results of these methods in their original articles.

*Soft-masked BERT* [8] masks the recognized erroneous characters and then hands over the processed input to the BERT model for error correction.

*PLOME* [27] pre-trains with misspelt knowledge for CSC.

*REALISE* [28] learns semantic, speech and visual representations through three encoders and integrates them using gating mechanisms.

*MDCSpell* [23] captures the visual and phonetic features and uses a post-fusion strategy to fuse the hidden state of the corrector and detector.

*LEAD* [29] constructs positive and negative samples based on the pronunciation, shape and definition knowledge in the dictionary.

*ECSpell* [15] combines glyph information with fine-grained phonological features and incorporates a dictionary-guided reasoning module.

*PGT* [12] utilizes a prior-knowledge guided teacher network to distill language modelling capabilities into the student model, mitigating overcorrection issues.

### 4.3. Experiment setting of large language models on the Chinese spelling correction task

**Figure 4.**
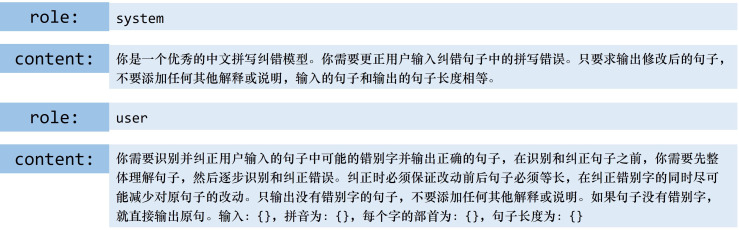
The prompt design.

We evaluate several of the latest LLMs, including *Qwen-max-0428, GLM-4-0520, Gemini-1.5-pro-exp-0801, Claude-3.5-sonnet-20240620 and GPT-4o-20240513* on the CSC task. The following experimental settings are employed:

*Prompt design*. To objectively and realistically evaluate the error-correction performance of LLMs on existing traditional datasets, we design the prompt as illustrated in figure 4.

*In-context learning strategies*. Previous studies have demonstrated that LLMs possess substantial in-context learning (ICL) capabilities, whereby their performance on specific tasks can be significantly improved through the provision of a limited number of task examples. Dong *et al.* [17] report that ChatGPT exhibits the most superior error correction performance under the 3-shot chain-of-thought strategy, significantly outperforming 0-shot, 1-shot and 5-shot paradigms based on systematic experimental evaluations. Empirical findings from [30] further indicate that model performance is strongly influenced by contextual diversity; the use of randomly sampled heterogeneous examples—including dialectal variations, domain-specific terminology and other complex error patterns—can effectively enhance generalization. Building upon these insights, this study proposes two differentiated sample selection strategies tailored to the unique characteristics of Chinese text error correction:

— Select a random error sample *(3-shot-random)*: randomly select three data points from the dataset.
— Select correct and wrong samples *(3-shot)*: choose three data points, including one sample with the correct original sentence and two samples with erroneous sentences. Specifically:
  - one sample with the correct original sentence to ensure that LLMs can maintain accurate input unchanged;
  - one sample with a phonetic similarity error to highlight the importance of phonetic-based corrections;
  - one sample with a shape error to emphasize the significance of shape-based corrections. The purpose of this design is to teach LLMs to correct errors while preserving correct input sentences and to demonstrate the importance of both phonetic and shape-based error types.

*Supervised instruction tuning*. As an open-source model specifically optimized for both Chinese and English, *ChatGLM3-6B* features a foundational architecture capable of accurately capturing the distinctive linguistic patterns inherent to the Chinese language, such as homophones and word order conventions. This enables its direct applicability to spelling correction tasks. Consequently, this model was selected for fine-tuning experiments within the scope of the study. To further explore its adaptability and effectiveness, two distinct fine-tuning strategies were adopted: *P-Tuning V2* and *LoRA*. The SIGHAN+WANG271K dataset is used for training. The structure of the instruction data is formulated as follows: (i) *task instructions:* modify the prompt to be more concise and consistent with user input; (ii) *input text:* the original sentence from the training data; (iii) *output text:* the corrected sentence.

### 4.4. Main results

The test results on the SIGHAN15 dataset are shown in table 1. Decopy achieves the highest precision and F1 score for both detection and correction tasks. Specifically, Decopy improves detection-level precision by 3.6% and correction-level precision by 4.5% compared with the previous best performance. Meanwhile, Decopy maintains a high recall rate, effectively balancing overcorrection and undercorrection.

**Table 1.** Sentence-level metrics for error detection and correction of each method on the SIGHAN15 test set. The bold values indicate the best performance on the corresponding metrics.

model	detection			correction		
	prec.	rec.	F1	prec.	rec.	F1
soft-masked BERT (2020)	73.7	73.2	73.5	66.7	66.2	66.4
PLOME (2021)	77.4	81.5	79.4	75.3	79.3	77.2
REALISE (2021)	77.3	81.3	79.3	75.9	79.9	77.8
MDCSpell (2022)	80.8	80.6	80.7	78.4	78.2	78.3
LEAD (2022)	79.2	82.8	80.9	77.6	81.2	79.3
ECSpell (2023)	81.1	**83.0**	81.0	77.5	**81.7**	79.5
PGT(BERT) (2024)	81.6	80.4	81.0	80.1	79.0	79.6
PGT(MDCSpell) (2024)	80.4	81.7	81.1	78.3	80.1	79.2
Decopy (ours)	**85.2**	79.0	**82.0**	**84.6**	75.1	**79.6**

It is worth noting that MDCSpell also employs a detection-correction framework but performs worse than Decopy. This inferior performance can be attributed to Decopy’s innovative error masking strategy, which effectively reduces the misdirection from incorrect characters. Compared with PLOME, a pre-training method, Decopy also demonstrates superior performance. This highlights the high quality of our custom pre-training dataset.

### 4.5. Domain specialization results

We further evaluate the three domain models on the test sets from ECSpell [15]. The results are shown in table 2. Decopy outperforms all other comparison methods on all test sets and significantly improves the optimal performance. Compared with ECSpell^UD^, a domain-adaptive CSC method, domain Decopy achieves F1 score improvements of 17.3, 13.5 and 13.5% on the detection level, and 16.1, 15.1 and 16.9% on the correction level. This demonstrates Decopy’s strong adaptability to specific language styles and terminologies. By leveraging an advanced detect-correct framework and robust pre-training, Decopy can perform the CSC task effectively on a variety of domain-specific texts.

**Table 2.** Sentence-level metrics for error detection and correction of each model on the LAW, medical treatment (Med) and official document writing (Odw) test sets. ‘^UD^’ denotes that the method is added with a user dictionary guided inference module [15] . The bold values indicate the best performance on the corresponding metrics.

model	detection			correction		
	prec	rec.	F1	prec	rec.	F1
**LAW**						
soft-masked BERT	53.5	48.3	50.8	38.4	34.7	36.5
soft-masked BERT^UD^	55.2	49.3	52.1	39.8	35.6	37.6
ECSpell	76.5	65.0	70.3	70.5	59.9	64.8
ECSpell^UD^	78.2	67.8	72.6	72.2	62.6	67.2
Decopy-law	**96.4**	**84.3**	**89.9**	**95.9**	**73.7**	**83.3**
**Med**						
soft-masked BERT	43.6	41.0	42.3	26.7	25.1	25.9
soft-masked BERT^UD^	44.4	45.1	44.7	26.8	29.2	28.0
ECSpell	75.2	60.6	67.1	67.3	54.2	60.0
ECSpell^UD^	75.8	65.8	70.4	68.0	58.6	62.8
Decopy-med	**88.6**	**79.6**	**83.9**	**87.3**	**70.3**	**77.9**
**Odw**						
soft-masked BERT	52.1	44.9	48.2	37.1	31.9	34.3
soft-masked BERT^UD^	53.4	49.0	51.1	37.5	35.1	36.3
ECSpell	81.4	63.6	71.4	76.3	59.6	67.0
ECSpell^UD^	82.4	70.1	75.8	76.9	64.3	69.2
Decopy-Odw	**96.0**	**83.5**	**89.3**	**95.7**	**78.2**	**86.1**

### 4.6. Effect of hyperparameter

The threshold theta of the error detection model is an important hyperparameter. The larger the theta, the stricter the error detection of the model. Table 3 shows the test results for the different thresholds. At a threshold of 0.72, Decopy achieves optimal performance on SIGHAN15, indicating a well-balanced approach between overcorrection and undercorrection. The optimal thresholds of the three domain models are 0.41, 0.21 and 0.48, respectively.

**Table 3.** Results under different θ. The bold values indicate the best performance on the corresponding metrics.

model	detection			correction		
	prec.	rec.	F1	prec.	rec.	F1
Decopy (θ=0.72)	85.2	**79.0**	**82.0**	84.6	**75.1**	**79.6**
Decopy (θ=0.6)	83.2	78.4	80.7	82.5	74.7	78.4
Decopy (θ=0.8)	**86.7**	76.2	81.1	**86.1**	72.3	78.6

### 4.7. Ablation study

To evaluate the effectiveness of our innovative work, we perform ablation experiments on SIGHAN15 in the following three aspects: (i) remove pinyin information from the correction network, (ii) remove the error masking strategy, and (iii) remove the pre-training.

The results of the ablation experiments are presented in table 4. Decopy without (w/o) pinyin information shows a decrease of 14.5% in the F1 score on the correction level. This indicates that the inclusion of pinyin information can significantly improve the correction ability of the model. Decopy w/o error masking strategy shows a decrease in the F1 score, especially on the correction level, up to 13%. On the detection level, despite a 0.6% increase in precision, the recall of the ablation model decreases by 3.3%, resulting in a worse F1 score. This demonstrates that the error masking strategy can effectively reduce misdirection from wrong characters. Decopy w/o pre-training shows a decrease of 4.9 and 8.3% in the F1 score on the two levels. This proves that pre-training enhances the models’ understanding of the input information.

**Table 4.** Results of the ablation study. The bold values indicate the best performance on the corresponding metrics.

model	detection			correction		
	prec.	rec.	F1	prec.	rec.	F1
Decopy	85.2	**79.0**	**82.0**	**84.6**	**75.1**	**79.6**
Decopy w/o pinyin information	**85.8**	75.7	80.4	75.9	56.9	65.1
Decopy w/o error masking strategy	85.3	76.1	80.4	81.1	56.2	66.4
Decopy w/o pre-training	79.9	74.9	77.1	77.8	65.9	71.3

### 4.8. Results of large language models on the Chinese spelling correction task

We test and evaluate the performance of several newer LLMs using the same evaluation metrics on SIGHAN15. The results are shown in table 5. It can be seen that despite careful constraints on the output of LLMs, their performance on all automatic evaluation indicators is still far lower than that of the Decopy model. To some extent, this phenomenon indicates that the CSC task remains very challenging for LLMs represented by GPT-4o.

**Table 5.** Results of LLMs and Decopy on SIGHAN15. The bold values indicate the best performance on the corresponding metrics.

model	detection			correction		
	prec.	rec.	F1	prec.	rec.	F1
Qwen-max-0428	21.69	35.92	27.05	18.15	30.06	22.63
GLM-4-0520	25.86	42.99	32.29	22.53	37.45	28.14
Gemini-1.5-pro-exp-0801	35.95	54.98	43.47	31.85	48.71	38.51
Claude-3.5-sonnet-20240620	38.95	60.52	47.40	35.15	54.61	42.77
GPT-4o-20240513	44.72	65.68	53.21	40.83	59.96	48.58
Decopy (ours)	**85.23**	**78.96**	**82.04**	**84.62**	**75.11**	**79.60**

Table 6 shows the results of the tests with *different context learning strategies* added to LLMs. The results show that the test input containing a small number of tag examples can enhance performance through context learning and is not limited to the selection strategies of the examples. This further proves the effectiveness of the context learning method. However, there is greater performance improvement when both correct and wrong samples are selected than when randomly wrong samples are selected. This reflects the impact of different context learning strategies on the model performance.

**Table 6.** Results of tests with different context learning strategies added to the LLMs. Select a random error sample (3-shot-random) and select correct and wrong samples (3-shot). The bold values indicate the best performance on the corresponding metrics.

model	detection			correction		
	prec.	rec.	F1	prec.	rec.	F1
GLM-4-0520	25.86	42.99	32.29	22.53	37.45	28.14
3-shot-random	33.49	**51.29**	40.52	28.67	**43.91**	34.69
3-shot	**36.60**	50.37	**42.39**	**30.97**	42.62	**35.87**
Claude-3.5-sonnet-20240620	38.95	60.52	47.40	35.15	54.61	42.77
3-shot-random	41.66	63.10	50.18	37.27	56.46	44.90
3-shot	**46.17**	**66.79**	**54.60**	**42.60**	**61.62**	**50.38**
GPT-4o-20240513	44.72	65.68	53.21	40.83	59.96	48.58
3-shot-random	46.31	70.66	55.95	42.81	65.31	51.72
3-shot	**52.35**	**73.99**	**61.31**	**47.52**	**67.16**	**55.66**

Table 7 shows the results of fine-tuning ChatGLM3-6B. We find that the LoRA fine-tuning method is obviously the most effective, significantly improving the performance of the model in various indicators, especially in the error detection task. This effectiveness can be attributed to the LoRA’s low-rank matrix adaptation approach, which retains the model’s original language capabilities while enhancing its adaptability to specific tasks. In contrast, although P-Tuning V2 contributes to improving model performance, it is not as effective as LoRA in the CSC task. This may be because P-Tuning V2 is better suited for scenarios that require extensive context understanding or cross-task fine-tuning, whereas LoRA’s direct parameter tuning is more effective for CSC tasks that require precise text processing and modification.

**Table 7.** Results of the fine-tuning on ChatGLM3-6B. Two fine-tuning strategies are used: P-Tuning V2 and LoRA . The bold values indicate the best performance on the corresponding metrics.

model	detection			correction		
	prec.	rec.	F1	prec.	rec.	F1
ChatGLM3-6B	8.63	7.75	8.08	6.83	6.27	6.54
LoRA	**32.20**	**36.53**	**34.23**	**25.20**	**28.60**	**26.79**
P-Tuning V2	12.54	14.02	13.24	9.90	11.07	10.45

## 5. Conclusion

This article proposes a new method called Decopy for CSC. First, inspired by the challenges in prior studies, we develop an advanced detection-correction framework with an innovative error masking strategy using pinyin features. Second, we construct a large, high-quality pre-training corpus for CSC and use it to pre-train Decopy. Experiments on SIGHAN15 and domain-specific datasets show that Decopy surpasses all comparison methods, achieving state-of-the-art performance and strong adaptability to domain-specific CSC. Additionally, we analyse the performance of several recent and powerful LLMs on the CSC task and find that it remains challenging for LLMs such as GPT-4. Future work will focus on integrating CSC methods with LLMs to enhance accuracy and efficiency, enabling better handling of complex natural language phenomena.

## Data Availability

Data and relevant code for this research work are stored in [31] and have been archived within the Zenodo repository in [32].
